# Dependence of the escape from an axially symmetric galaxy on the energy

**DOI:** 10.1038/s41598-021-87670-5

**Published:** 2021-04-19

**Authors:** Juan F. Navarro

**Affiliations:** Department of Applied Mathematics, University of Alicante, 03690 Alicante, Spain

**Keywords:** Astronomy and astrophysics, Applied mathematics, Astronomy and planetary science

## Abstract

The escape of a particle from a dynamical system depends on the intersection between the ingoing and outgoing asymptotic trajectories to certain periodic orbits placed at the openings of the curves of zero velocity of the system. Although many efforts have been devoted to the analysis of the escape from potentials presenting multiple openings, there are still few studies on potentials with only one opening. In this article, we clarify the way in which the energy affects the escape in this type of systems, showing that, contrary to what one could expect, there are several bifurcations for certain values of the energy.

## Introduction

The motion of a particle in a gravitational potential and its possibility of escape from it has been extensively studied^1–20^. In these systems, the motion of the particle is limited by the curves of zero velocity. These curves, for certain values of the energy of the system, open creating windows, and particles can leave the system through them. There is one unstable periodic orbit, called Lyapunov orbit, located at any of the openings of the curves of zero velocity of the system. If the test particle crosses one of these orbits, then the particle leaves the potential well. In particular, the escape of a particle from a system depends on the relation of its initial conditions with respect to the stable manifolds to the Lyapunov orbits. If the initial conditions belong to the inner part of these structures, then the orbit will escape and, if not, it will remain trapped forever.

Most of the works devoted to escapes from dynamical systems analyze systems that present multiple openings. In these systems, the way in which the escape occurs depends on the energy of the system, since a variation in its value makes the intersections of the asymptotic curves of the different Lyapunov orbits vary considerably. However, few works have been dedicated to the study of systems with only one exit channel. We think that this type of potentials have the property of showing in a simple way the logic that explains the escape of a particle from the system. It remains to be studied if the variation of the energy produces a variation in the form in which the escape takes place in this type of systems. The object of this paper is to clarify this question.

To this end, we study the escape from a galactic model with axial symmetry. This type of galactic system has been previously studied by Zotos^19^ and Navarro^12,13^. In a companion paper, Navarro^13^ investigates the geometry of the curves that delimite the escape domains by determining the intersection of the ingoing and outgoing asymptotic trajectories to the Lyapunov orbit with an apropiate surface of section, for a fixed value of the energy. In this paper, we describe how these limiting curves evolve as the energy of the system varies, showing that the intersection between the ingoing and outgoing asymptotic trajectories to the Lyapunov orbit takes place in a way that relies on the energy of the system.

## The equations of motion

We analyze the motion of a particle in the (*r*, *z*) meridian plane near the central part of an axially symmetric galaxy modeled by a galactic type potential of the form

1 $$\begin{aligned} V(r,z) = \frac{\omega ^2}{2} (r^2+z^2) -\mu \left( \alpha (r^4+z^4)+2\rho r^2z^2\right) \,, \end{aligned}$$

which is made up of perturbed harmonic oscillators with frequency $\omega$ along the *r* and *z* axis, where *r* and *z* are cylindrical coordinates, $\mu$ is the perturbation strength and $\alpha$ and $\rho$ are parameters^13,19^. The potential (1) can be derived by expanding global galactic potentials near the central stable equilibrium point of the system, located at the center of the galaxy. This type of potentials appears when the density distribution near the center of the galaxy is an analytic function of the coordinates and the Taylor series for the corresponding potential is truncated at fourth order. We must remark at this point that our galactic potential is truncated at $r_{max}= 1.5$ kpc, otherwise the mass density increases outwards from the center, a fact that is practically never observed in galaxies.

As *V*(*r*, *z*) is axially symmetric and the $L_z$ component of the angular momentum is conserved, the dynamical structure of the galactic system can be described by the effective potential^19^

2 $$\begin{aligned} W(r,z) = \frac{L_z^2}{2r^2} + V(r,z)\,, \end{aligned}$$

and the equations of motion are

3 $$\begin{aligned} \ddot{r}&= -\frac{\partial W}{\partial r} = -\omega ^2 r +4\mu \alpha r^3 +4\mu \rho rz^2 + \frac{L_z^2}{r^3}\,, \\ \ddot{z}&= -\frac{\partial W}{\partial z} = -\omega ^2 z +4\mu \alpha z^3 + 4\mu \rho r^2z\,. \end{aligned}$$

The Hamiltonian corresponding to the effective potential given by Eq. (2) is

4 $$\begin{aligned} H = \frac{1}{2}({\dot{r}}^2+{\dot{z}}^2) + \frac{\omega ^2}{2} (r^2+z^2) -\mu \left( \alpha (r^4+z^4)+2\rho r^2z^2\right) + \frac{L_z^2}{2r^2}\,, \end{aligned}$$

where *H* is the numerical value of the energy of the system, which is conserved. We can obtain the curves of zero velocity by substituting ${\dot{r}}={\dot{z}}=0$ into Eq. (4), to get

5 $$\begin{aligned} H= \frac{L_z^2}{2r^2} + \frac{\omega ^2}{2} (r^2+z^2) -\mu \left( \alpha (r^4+z^4)+2\rho r^2z^2\right) \,. \end{aligned}$$

For small values of the energy of the system, the curves of zero velocity are closed curves, and test particles can not escape from the system. But there exists a value of the energy, known as energy of escape and denoted by $H_c$, such that if the energy of the test particle exceeds $H_c$, then the curves of zero velocity open at one place and particles may escape from the system. For those values of *H*, there is an unstable periodic orbit at the exit of the potential.

**Figure 1:**
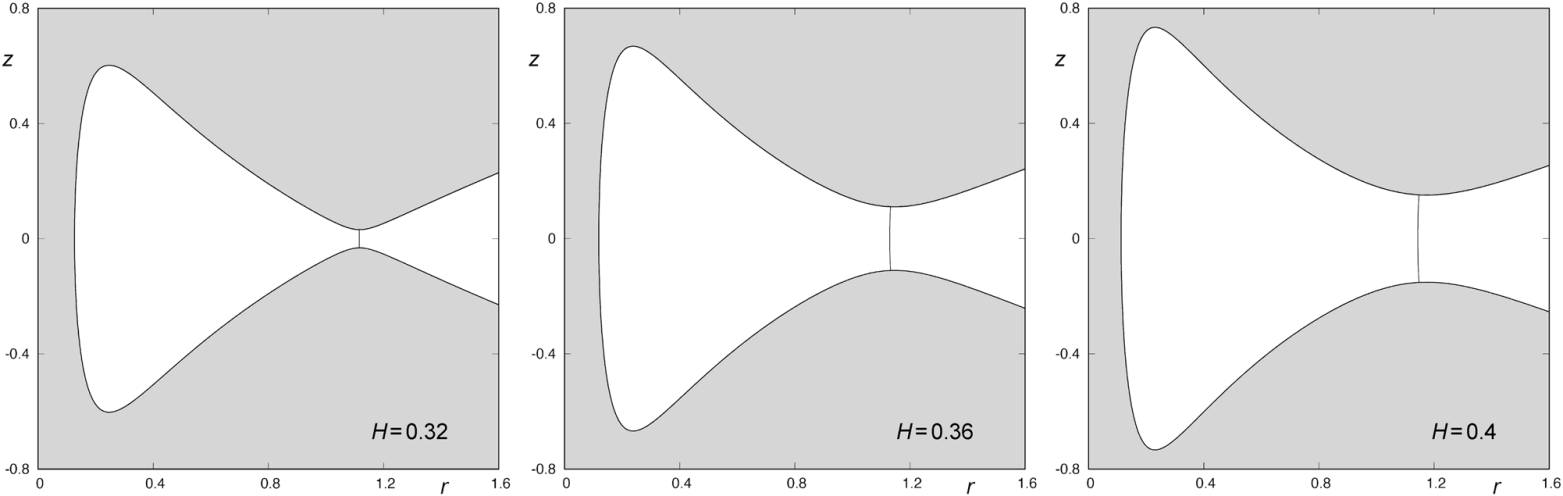
Curves of zero velocity for $\omega =1$, $\mu =1$, $\alpha =0.2$, $\rho =-1.2$, $L_z=0.1$ and $H_1=0.32$ (left panel), $H_2=0.36$ and $H_3=0.4$ (right panel). The almost straight line barring the opening of the potential is the Lyapunov orbit. This graphic has been generated by using Gnuplot Version 5.2. http://www.gnuplot.info.

In our work, we use a system of galactic units, where the unit of length is 1 kpc, the unit of time is $10^7$ yr, the unit of velocity is ($1\hbox { kpc})/(10^7\hbox { yr}) = 97.8\hbox { km/s}$, and the unit of energy is $1\hbox { kpc}^2/(10^7\hbox { yr})^2$. Throughout this paper, we have taken the following values for the parameters: $\omega =1$ $(10^7\hbox { yr})^{-1}$, $\mu =1$ $(10^7\hbox { yr kpc})^{-2}$, $\alpha =0.2$, $\rho =-1.2$ and $L_z=0.1$. Then, the energy of escape is given by $H_c = 0.3125$^13^. In Fig. 1, we show the curves of zero velocity for some values of the energy larger than $H_c$, as well as the Lyapunov orbit “guarding” the escape from the system. As the value of the energy of the system grows, the size of the opening becomes bigger.

## Analysis of the asymptotic trajectories to the Lyapunov orbit

As we have stated above, the asymptotic trajectories to the Lyapunov orbit constitute the limit of the set of escaping orbits. It is common to use an adequate surface of section in order to unveil the geometry of these complex sets by analyzing their successive intersections on it. These crossings between the stable manifold to the Lyapunov orbit and the surface of section define the contour of the basins of escape, that is, the set of initial conditions leading to escape. For the computation of the initial part of these asymptotic curves, we follow the method suggested by Deprit and Henrard^21^. The selection of the initial conditions of these curves have been determined by following the procedure described by Navarro^13^. We have computed and integrated backward a set of $1\,000\,000$ initial conditions taken in the ingoing asymptotic orbits to the Lyapunov orbit, until they intersect the surface of section $r={\bar{r}}$ (${\dot{r}}>0$), where ${\bar{r}}$ is an adequate constant value. For our numerical exploration, we have taken ${\bar{r}}=0.6$.

In the following, $\phi$ denotes the periodic orbit placed at the exit channel, $W_{s,\nu }(\phi )$ denotes the $\nu$-th intersection between the ingoing asymptotic trajectories to $\phi$ and the hyperplane $r={\bar{r}}$, and $W_{u,\nu }(\phi )$ the $\nu$-th intersection of the outgoing asymptotic trajectories with $r={\bar{r}}$. Here, $\nu \in {\mathbb {N}}$. We have calculated these structures for a sequence of values of the energy given by $H=H_0+n \Delta H$, where $H_0=0.32$, $\Delta H=0.0001$ and $n\in {\mathbb {N}}_0$, $0\le n \le 800$. For all these values, we have computed the sets $W_{s,\nu }(\phi )$, for $\nu =1,2,3,4$, and $W_{u,1}(\phi )$. For any value of the energy considered, we have

$$\begin{aligned} W_{s,1}(\phi ) \cap W_{u,1}(\phi )=\emptyset \,, \end{aligned}$$

as $W_{u,1}(\phi )$ takes place for ${\dot{r}}<0$, while $W_{s,1}(\phi )$ for ${\dot{r}}>0$. Orbits with initial conditions in the region bounded by $W_{s,1}(\phi )$ straightaway leave the potential well through the exit channel, while initial conditions inside the domain enclosed by $W_{u,1}(\phi )$ correspond to orbits coming from the infinity.

**Figure 2:**
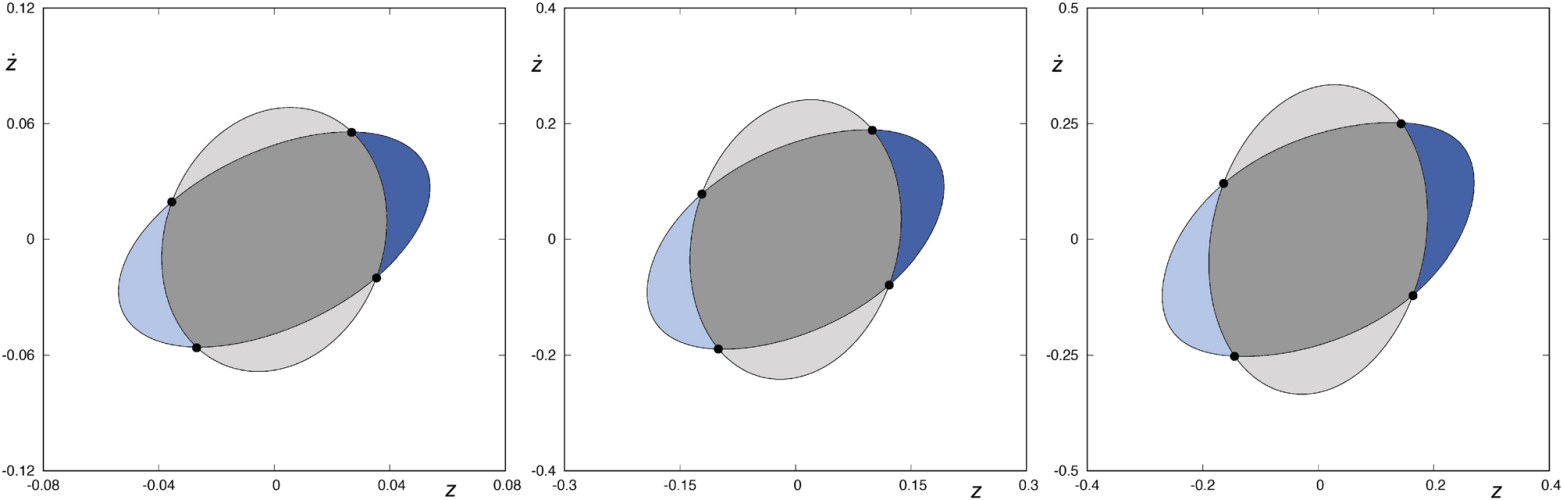
Intersection between $W_{s,2}(\phi )$ and $W_{u,1}(\phi )$ for $H=0.32, 0.36$ and 0.44 (from left to right, respectively). Orbits starting in the area enclosed by $W_{u,1}(\phi )$, colored in light or dark grey, come from the infinity. Orbits with initial conditions belonging to the area enclosed by $W_{s,2}(\phi )$ and $W_{u,1}(\phi )$, colored in a dark grey tone, come from the infinity and leave the galaxy after intersecting the hyperplane $r={\bar{r}}$ two times. Orbits starting in the area delimited by one of the pair of tongues colored in blue can be integrated backward until they intersect again the hyperplane $r={\bar{r}}$, and leave the galaxy in the future by the opening of the curves of zero velocity. This graphic has been generated by using Gnuplot Version 5.2. http://www.gnuplot.info.

The sets $W_{s,2}(\phi )$ and $W_{u,1}(\phi )$ (both with ${\dot{r}}<0$) are shown in Fig. 2, for several values of the energy of the system ($H= 0.32, 0.36$ and 0.4). We can observe that these sets have four points in common, marked with black dots in Fig.  2, belonging to four homoclinics to the Lyapunov orbit. Each of these homoclinics intersects the hyperplane defined by $r={\bar{r}}$ at two different instants of time^13^. Orbits with initial conditions belonging to the area enclosed by $W_{s,2}(\phi )$ and $W_{u,1}(\phi )$, colored in a dark grey tone in Fig. 2, come from the infinity and leave the galaxy after intersecting the hyperplane $r={\bar{r}}$ two times. Furthermore, orbits starting in the area delimited by one of the pair of “tongues”, colored in blue in Fig. 2, can be integrated backward until they intersect again the hyperplane $r={\bar{r}}$, and leave the galaxy in the future by the opening of the curves of zero velocity.

**Figure 3:**
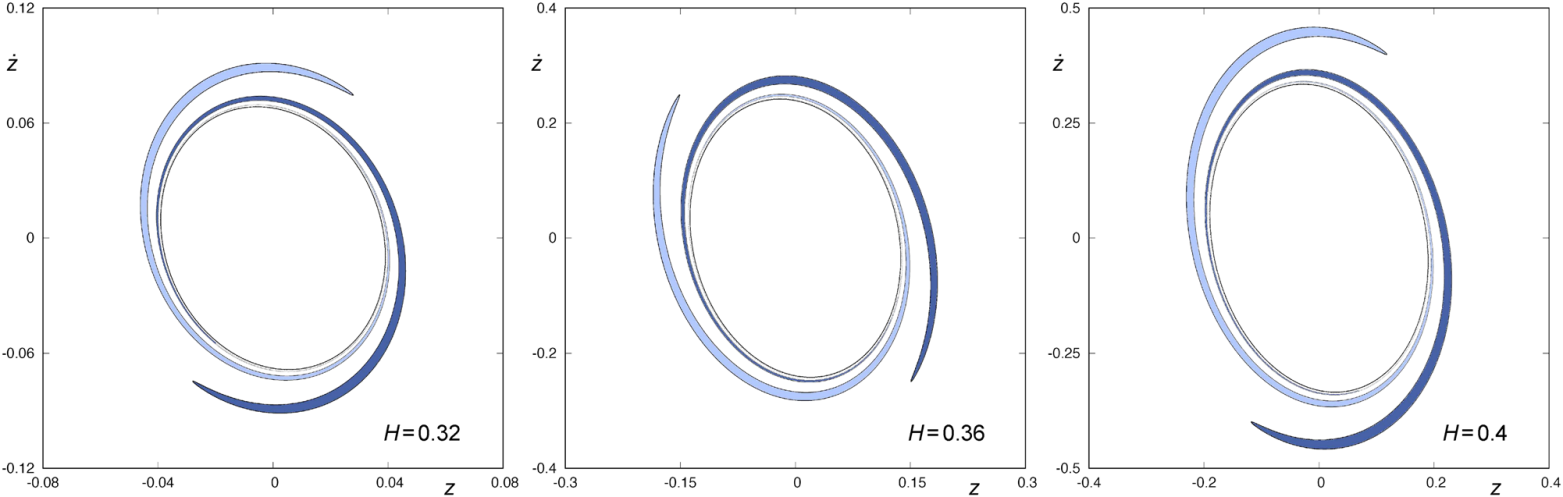
$W_{s,3}(\phi )$ for $H=0.32, 0.36$ and 0.4 (from left to right, respectively). The area enclosed by $W_{s,3}(\phi )$ is colored in light and dark blue. These two blue colored infinite tongues are the result of the backward integration of the tongues colored in the same shade of blue in Fig. 2. This graphic has been generated by using Gnuplot Version 5.2. http://www.gnuplot.info.

**Figure 4:**
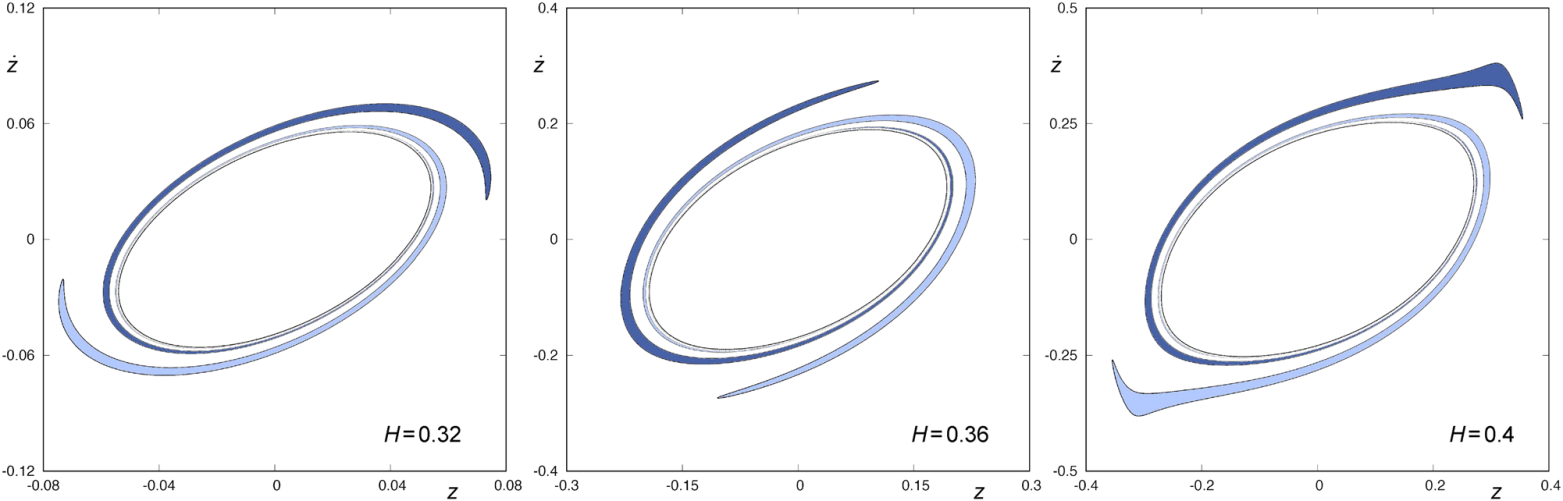
$W_{s,4}(\phi )$ for $H=0.32, 0.36$ and 0.4 (from left to right, respectively). The area enclosed by $W_{s,4}(\phi )$ is colored in blue. The two infinite tongues colored in light and dark blue are the result of the backward integration of the tongues colored in the same shade of blue in Fig. 3. This graphic has been generated by using Gnuplot Version 5.2. http://www.gnuplot.info.

Thus, we can integrate backward the two tongues colored in blue in Fig. 2 up to their next intersection with the surface of section $r={\bar{r}}$, to obtain $W_{s,3}(\phi )$. These sets are shown in Fig. 3 for $H=0.32, 0.36$ and 0.4. We can observe that, for any of the values of *H* considered, $W_{s,3}(\phi )$ is made up of two tongues that rotate infinitely around $W_{s,1}(\phi )$. Each of these infinite tongues is the result of the backward integration of the tongues colored in the same shade of blue in Fig. 2. $W_{s,3}(\phi )$ does not intersect $W_{u,1}(\phi )$, as both sets have a different sign for ${\dot{r}}$. As a result, orbits starting inside one of these two infinite tongues have a preceding intersection with the surface of section, and the structure of the fourth crossing of the ingoing asymptotic trajectories to $\phi$ with the hyperplane $r={\bar{r}}$ is the same as that of $W_{s,3}(\phi )$, that is, is composed of a pair of tongues infinitely spiraling around $W_{s,2}(\phi )$. In Fig.  4, we depict $W_{s,4}(\phi )$ for $H=0.32,0.36$ and 0.4. As with the third intersection, orbits starting in the area delimited by one of these two infinite tongues leave the central part of the galaxy through the opening of the potential well, after intersecting the surface of section $r={\bar{r}}$ at three points.

**Figure 5:**
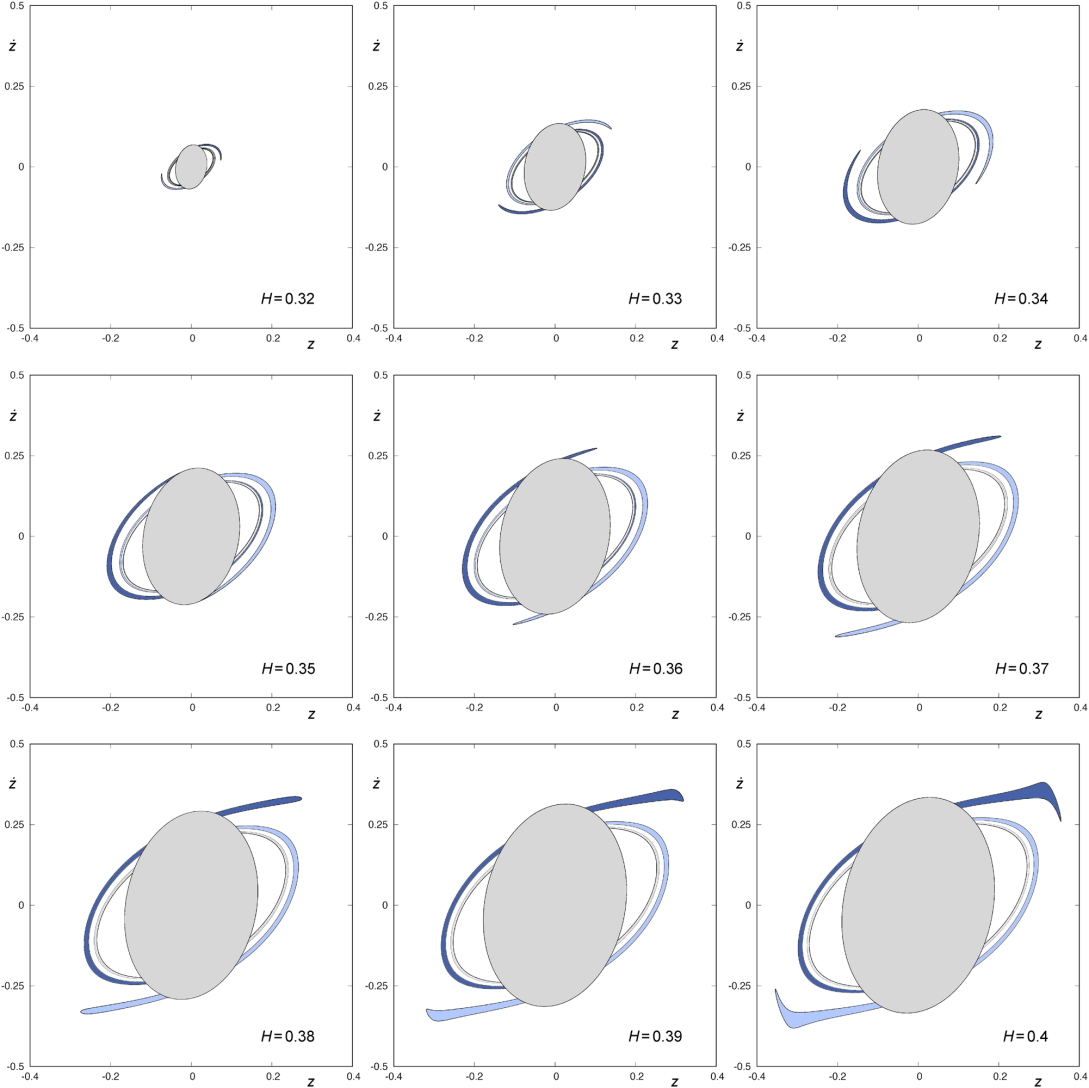
$W_{s,4}(\phi )$ and $W_{u,1}(\phi )$ for $H=H_0 + n\Delta H$, where $H_0=0.32$, $\Delta H=0.01$ and $0\le n \le 8$. Orbits starting in the area enclosed by $W_{u,1}(\phi )$ are colored in light grey. Orbits with initial conditions belonging to the area enclosed by $W_{s,4}(\phi )$ and outside the area enclosed by $W_{u,1}(\phi )$ are colored in blue. This graphic has been generated by using Gnuplot Version 5.2. http://www.gnuplot.info.

Contrary to what happens with $W_{s, 3}(\phi )$, $W_{s,4}(\phi )$ does intersect $W_{u,1}(\phi )$. In order to unveil the mode in which it does and analyze the concurrence of some bifurcation in the way this intersection takes place, we show in Fig. 5 a sequence of joint representations of the sets $W_{s,4}(\phi )$ and $W_{u,1}(\phi )$, for values of the energy given by $H=H_0 + n \Delta H$, where $H_0 = 0.32$, $\Delta H = 0.01$ and $0 \le n \le 8$. In these graphs, we have colored the interior of $W_{u,1}(\phi )$ in light gray. To show the variation in both the shape and the size of the windows, we have represented all the sets using the same framework: $z \in [-0.4,0.4]$ and ${\dot{z}} \in [-0.5,0.5]$. In Fig. 5, we can observe that the way $W_{s,4}(\phi )$ intersects $W_{u,1} (\phi )$ is the same in the most of the cases: in the part of $W_{s,4}(\phi )$ that stays outside the area enclosed by $W_{u,1}(\phi )$, we find a pair of tongues and a pair of sequences of “bridges”, as it has been described in detail by Navarro^13^. However, we can clearly observe that for a range of values of the energy in a neighborhood of $H = 0.35$, the pair of tongues disappears, since they remain contained within the area enclosed by $W_ {u,1}(\phi )$ and, therefore, we only find, outside $W_{u,1}(\phi )$, a pair of sequences of bridges. In fact, there is also a range of energy values in a neighborhood of $H = 0.323$ in which the same occurs, although it can not be appreciated in the sequence given in Fig. 5.

Orbits starting in the area delimited by $W_{u,1}(\phi )$ and one of the two infinite tongues of $W_{s,4}(\phi )$ enter the central part of the galaxy from the infinity and, after intersecting the surface of section $r={\bar{r}}$ at four points, leave the potential well.

Next, we will show how the hiding of the “tip” of the infinite tongues that make up $W_{s,4}(\phi )$ occurs, for values of *H* near $H = 0.323$ and $H = 0.35$. To that end, we have analyzed the intersection between $W_{s,4}(\phi )$ and $W_{u,1}(\phi )$ for values of the energy given by $H=H_0+n \Delta H$, where $H_0=0.32$, $\Delta H=0.0001$ and $n\in {\mathbb {N}}_0$, $0\le n \le 800$. The conclusions of this numerical exploration are described in the following paragraphs.

**Figure 6:**
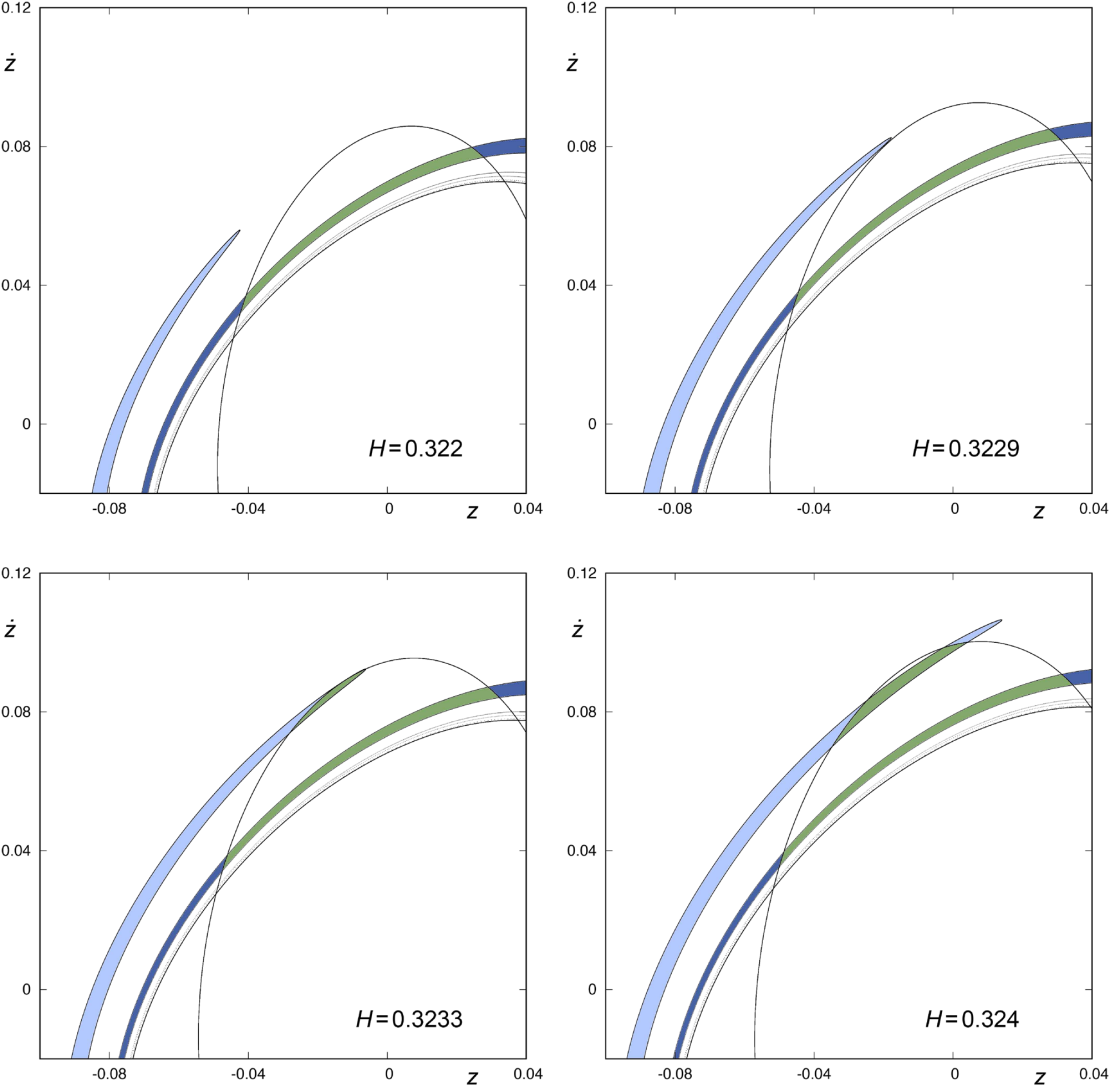
Detail of $W_{s,4}(\phi )$ and $W_{u,1}(\phi )$ for $H=0.322, 0.3229, 0.3233$ and 0.324. The area enclosed by $W_{s,4}(\phi )$ is colored in green or blue, depending on whether it is contained in the area enclosed by $W_{u,1}(\phi )$ or not, respectively. Orbits starting in the green colored area enter the central part of the galaxy from the infinity and, after intersecting the surface of section $r={\bar{r}}$ at four points, leave the potential well. This graphic has been generated by using Gnuplot Version 5.2. http://www.gnuplot.info.

In Fig. 6, we show a detail of $W_{s,4}(\phi )$ and $W_{u,1}(\phi )$, for values of the energy given by $H=0.322$, 0.3229, 0.3233 and 0.324, in order to unveil the way in which the intersection between these sets takes place. In the upper-left panel of Fig. 6, we can observe that the tip of one of the tongues belonging to $W_{s,4}(\phi )$, colored in light blue, is not contained in the region defined by $W_{u,1}(\phi )$, when $H=0.322$. If we examine the evolution of the tongues as the energy grows, we find that there exists a value ${H}_{\nu ,1}$ of the energy, slightly larger than $H=0.3229$, such that the tip of each of the infinite tongues that make up $W_{s,4}(\phi )$ is tangent to $W_{u,1}(\phi )$, that is, the tip of each tongue intersects $W_{u,1}(\phi )$ at just one point. In the upper-right panel of Fig. 6, we observe how the tip of one of the tongues belonging to $W_{s,4}(\phi )$, colored in light blue, is very close to be tangent to $W_{u,1}(\phi )$. If the value of the energy is slightly larger than $H_{\nu ,1}$, the tip of each tongue enters the region enclosed by $W_{u,1}(\phi )$, as depicted in the lower-left panel of Fig. 6. There is a value ${H}_{\nu ,2}$ of the energy, barely smaller than $H=0.3233$, such that the tip of each tongue of $W_{s,4}(\phi )$, now contained in the area enclosed by $W_{u,1}(\phi )$, is tangent to $W_{u,1}(\phi )$ at one point. For values of the energy slightly larger than ${H}_{\nu ,2}$, the tip of each tongue goes out of the region defined by $W_{u,1}(\phi )$, as we can observe in the lower-right panel of Fig. 6. Therefore, for values of the energy in the interval $I_\nu = ({H}_{\nu ,1}, {H}_{\nu ,2})$, the tip of each tongue belonging to $W_{s,4}(\phi )$ is contained in the area enclosed by $W_{u,1}(\phi )$ and, consequently, the area delimited by these two sets (the tip of each tongue and $W_{u,1}(\phi )$) corresponds to initial conditions of orbits coming from the infinity and escaping from the galaxy after intersecting the surface of section at four different points. In the same way, for values of the energy that are not in the interval $I_\nu$, the tip of each of the infinite tongues that make up $W_{s,4}(\phi )$ is outside the area enclosed by $W_{u,1}(\phi )$, so the initial conditions inside this part of the tongue have an antecedent, that is, we can integrate these initial conditions backward up to the fifth intersection.

**Figure 7:**
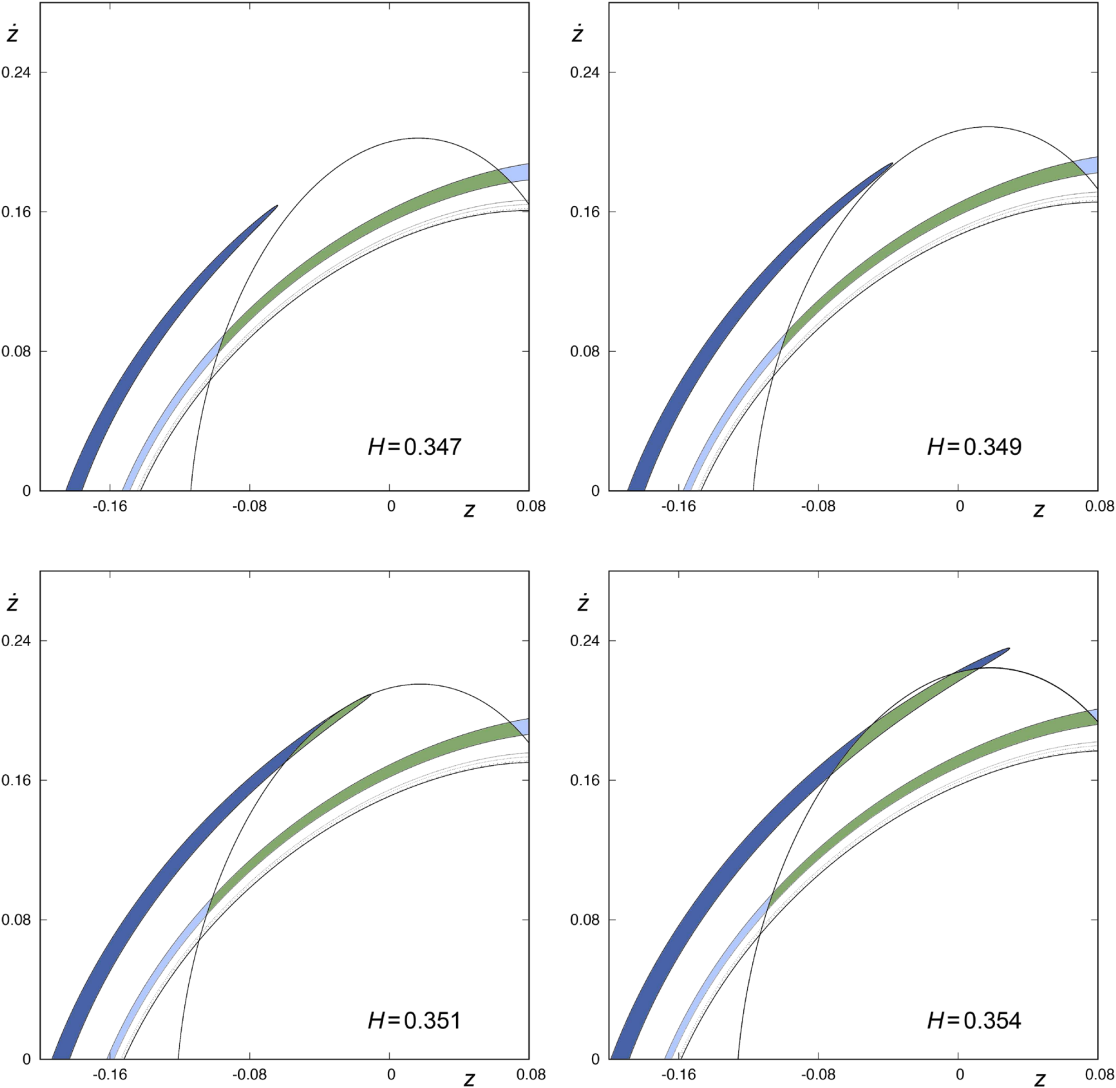
Detail of $W_{s,4}(\phi )$ and $W_{u,1}(\phi )$ for $H=0.347, 0.349, 0.351$ and 0.354. The area enclosed by $W_{s,4}(\phi )$ is colored in green or blue, depending on whether it is contained in the area enclosed by $W_{u,1}(\phi )$ or not, respectively. Orbits starting in the green colored area enter the central part of the galaxy from the infinity and, after intersecting the surface of section $r={\bar{r}}$ at four points, leave the potential well. This graphic has been generated by using Gnuplot Version 5.2. http://www.gnuplot.info.

We observe this same phenomenon for values of *H* in a neighborhood of $H=0.35$. In Fig. 7, we show a detail of $W_{s,4}(\phi )$ and $W_{u,1}(\phi )$, for values of the energy given by $H=0.347$, 0.349, 0.351 and 0.354. In the upper-left panel of Fig. 7, we can observe that the tip of one of the tongues belonging to $W_{s,4}(\phi )$, colored in dark blue, is not contained in the region defined by $W_{u,1}(\phi )$, when $H=0.347$. If we examine the evolution of the tongues as the energy grows, we find that there exists a value ${H}_{\alpha ,1}$ of the energy, slightly larger than $H=0.349$, such that the tip of each of the infinite tongues that make up $W_{s,4}(\phi )$ is tangent to $W_{u,1}(\phi )$, that is, the tip of each tongue intersects $W_{u,1}(\phi )$ at just one point. In the upper-right panel of Fig. 7, we observe how the tip of one of the tongues belonging to $W_{s,4}(\phi )$, colored in dark blue, is very close to be tangent to $W_{u,1}(\phi )$. If the value of the energy is slightly increased over $H_{\alpha ,1}$, the tip of each tongue belonging to $W_{s,4}(\phi )$ enters the region enclosed by $W_{u,1}(\phi )$, as depicted in the lower-left panel of Fig. 7. There is a value ${H}_{\alpha ,2}$ of the energy, barely smaller than $H=0.351$, such that the tip of each tongue of $W_{s,4}(\phi )$, now contained in the area enclosed by $W_{u,1}(\phi )$, is tangent to $W_{u,1}(\phi )$ at one point. For values of *H* slightly larger than ${H}_{\alpha ,2}$, the tip of each tongue belonging to $W_{s,4}(\phi )$ goes out of the region defined by $W_{u,1}(\phi )$, as we can observe in the lower-right panel of Fig. 7. Therefore, for values of the energy in the interval $I_\alpha = ({H}_{\alpha ,1}, {H}_{\alpha ,2})$, the tip of each tongue belonging to $W_{s,4}(\phi )$ is contained in the area enclosed by $W_{u,1}(\phi )$ and, consequently, the area delimited by these two sets (the tip of each tongue and $W_{u,1}(\phi )$) corresponds to initial conditions of orbits coming from the infinity and escaping from the galaxy after intersecting the surface of section at four different points. In the same way, for values of the energy that are not in the interval $I_\alpha$, the tip of each of the infinite tongues that make up $W_{s,4}(\phi )$ is outside the area enclosed by $W_{u,1}(\phi )$, so the initial conditions inside this part of the tongues have an antecedent, that is, we can integrate these initial conditions backward up to obtain $W_{s,5}(\phi )$.

**Figure 8:**
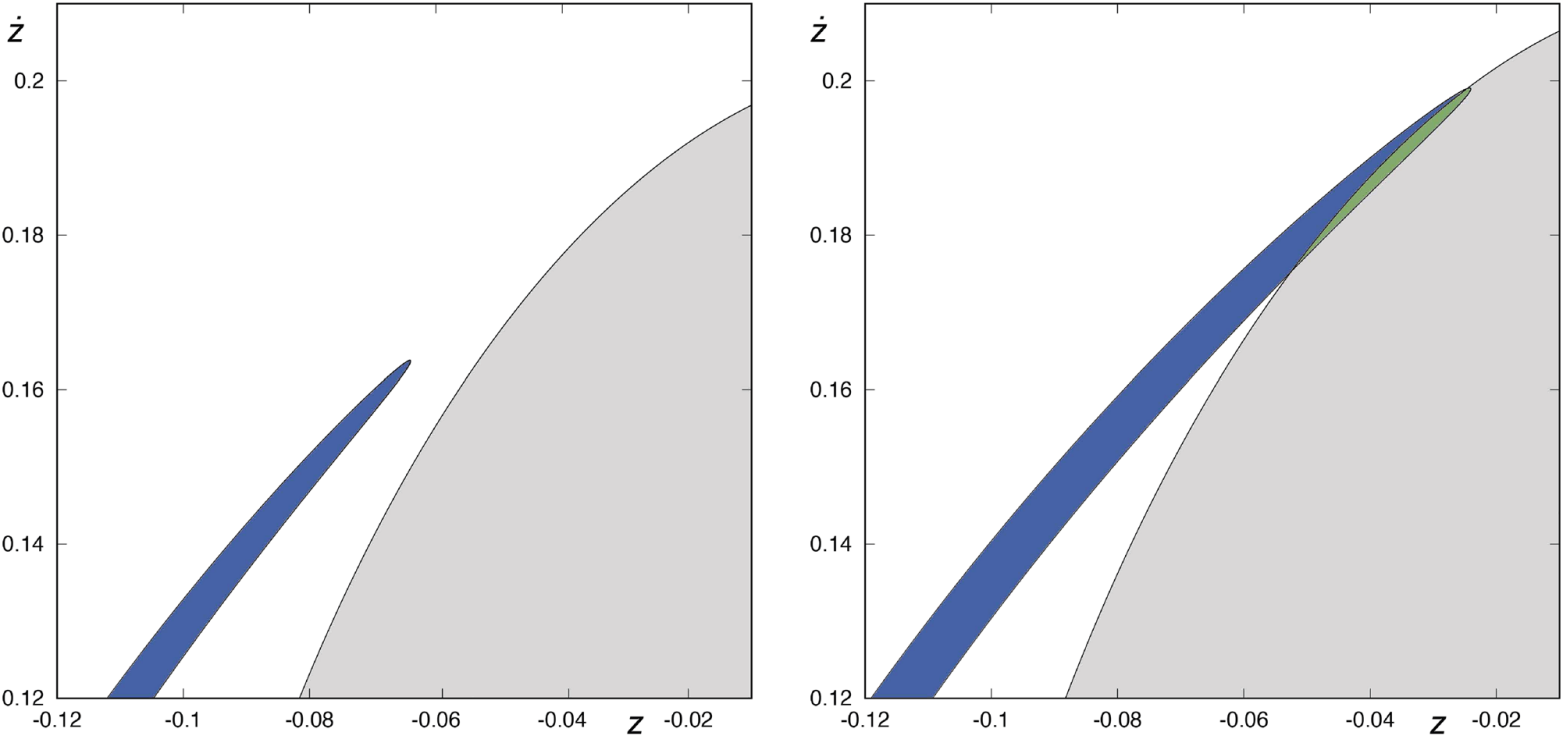
Tip of one of the tongues belonging to $W_{s,4}(\phi )$, for $H=0.347$ (left panel) and $H=0.35$ (right panel). The area enclosed by $W_{s,4}(\phi )$ is colored in green or blue, depending on whether it is contained in the area enclosed by $W_{u,1}(\phi )$ or not, respectively. This graphic has been generated by using Gnuplot Version 5.2. http://www.gnuplot.info.

Thus, we can conclude that we can find two different scenarios, both depicted in Fig. 8. Let us define $I= I_\nu \cup I_\alpha$.

1. If $H\not \in I$, the part of $W_{s,4}(\phi )$ that stays outside the area enclosed by $W_{u,1}(\phi )$ is composed of a pair of infinite sequences of bridges and a pair of tongues. In the left panel of Fig. 8, we show the tip of one of the tongues that make up $W_{s,4}(\phi )$, colored in blue, for $H=0.347$.
2. If $H\in I$, the part of $W_{s,4}(\phi )$ outside the area enclosed by $W_{u,1}(\phi )$ is defined by a pair of infinite sequences of bridges. In this case, the tip of each of the tongues belonging to $W_{s,4}(\phi )$ is contained in the area enclosed by $W_{u,1}(\phi )$, so the tip of the tongue ends the infinite chain of bridges, as it can be observed in the right panel of Fig.  8. We have colored in blue the bridge that ends the sequence of bridges and, in green, the part of the tongue contained in the area enclosed by $W_{u,1}(\phi )$.

Therefore, depending on whether $H \in I$ or not, the part of $W_{s,4}(\phi )$ that have an antecedent on the surface of section if we integrate backward is different.

Finally, we observe that when we increase the energy of the system, the amount of orbits that escape varies substantially. To show this, we have calculated the percentage of orbits that escape directly without intersecting the surface of section, by considering a mesh of $1024 \times 1024$ equally spaced initial conditions belonging to the surface of section $r = {\bar{r}}$, ${\dot{r}} > 0$, and enclosed by the limit curve defined by

6 $$\begin{aligned} 2H = {\dot{z}}^2 + \omega ^2({\bar{r}}^2+z^2) -2\mu \left( \alpha ({\bar{r}}^4+z^4) +2 \rho {\bar{r}}^2 z^2\right) + L_z^2/{\bar{r}}^2 \end{aligned}$$

in the $(z, {\dot{z}})$ plane. The integration has been carried out up to a maximum time of 6 units of time, to ensure that the initial conditions escape directly without crossing the surface of section, due to the fact that the time at which the first intersection of the ingoing asymptotic trajectories to $\phi$ with the surface of section occurs never exceeds 5 units of time for any of the values of the energy considered. Moreover, the following intersection of the ingoing asymptotic trajectories with the hyperplane $r={\bar{r}}$, that is, $W_{s,2}(\phi )$, takes place for a time of integration larger than 7 units of time.

**Figure 9:**
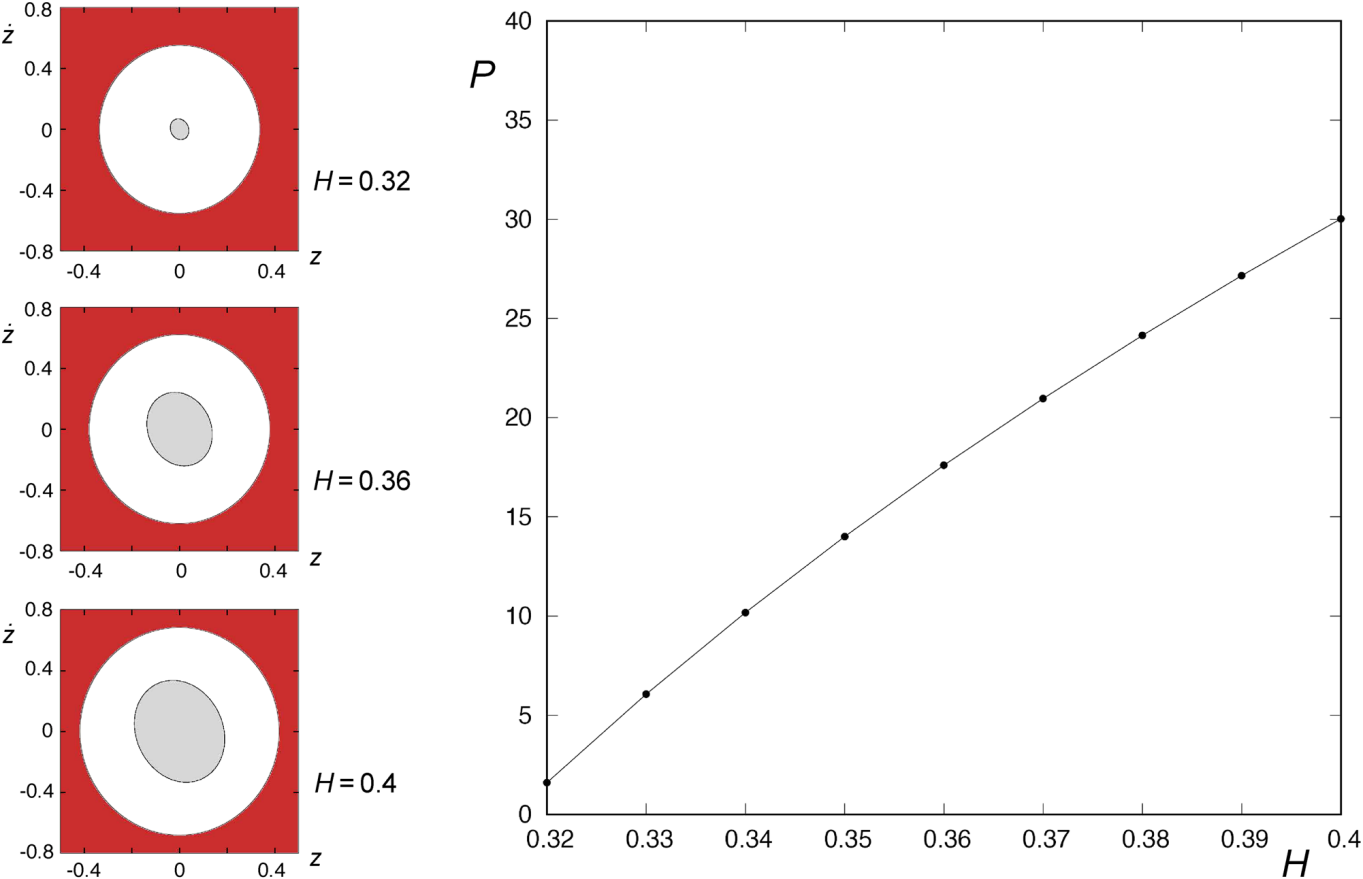
In the right panel, percentage of area that the region enclosed by $W_{s,1}(\phi )$ occupies in the domain bounded by the limit curve (6), for values of the energy given by $H=H_0 + n\Delta$, where $H_0 = 0.32$, $\Delta H = 0.01$ and $0 \le n \le 8$. In the three panels in the left, we represent $W_{s,1}(\phi )$ together with the limit curve, for $H = 0.32,0.36$ and 0.4 (from the top to the bottom, respectively). The initial conditions of orbits colored in grey correspond to orbits that escape directly from the potential well. We have marked the set of initial conditions outside the limit curve, and prohibited by the value of energy, in red color. This graphic has been generated by using Gnuplot Version 5.2. http://www.gnuplot.info.

In the right panel of Fig.  9, we show the percentage of area that the region enclosed by $W_{s,1}(\phi )$ occupies in the domain bounded by the limit curve described in Eq.  (6), for values of the energy given by $H=H_0 + n\Delta$, where $H_0 = 0.32$, $\Delta H = 0.01$ and $0 \le n \le 8$. In the three panels in the left of Fig. 9, we represent $W_{s,1}(\phi )$ together with the limit curve given by (6), for $H = 0.32,0.36$ and 0.4 (from the top to the bottom, respectively). The initial conditions of orbits colored in grey correspond to orbits that escape directly from the potential well. We have marked the set of initial conditions outside the limit curve, and prohibited by the value of energy, in red color.

## Conclusions

The study of escapes in dynamical systems presenting one sole exit channel is a topic that has not received much attention by the scientific community. However, we think that these systems can exhibit very clearly the logic that explains the escape of a particle from the system. In this paper, we analyze this question, clarifying if the variation of the energy produces a variation in the way in which the escape takes place in this type of systems. To this end, we have studied the escape from a galactic model with axial symmetry, by investigating the geometry of the limiting curves of the escape domains by determining the intersection of the ingoing and outgoing asymptotic trajectories to the Lyapunov orbit with the surface of section. We have shown that, in fact, there are two intervals of values of the energy where the intersection between the ingoing and the outgoing asymptotic trajectories to the Lyapunov orbit takes place in a different way.
